# Acute Onset Focal Epilepsy Mimicking Stroke

**DOI:** 10.7759/cureus.18600

**Published:** 2021-10-08

**Authors:** Marilena Mangiardi, Sabrina Anticoli, Luca Bertaccini, Valeria Cozzolino, Francesca Romana Pezzella

**Affiliations:** 1 Stroke Unit, Azienda Ospedaliera San Camillo Forlanini, Rome, ITA; 2 Neuroradiology, Azienda Ospedaliera San Camillo Forlanini, Rome, ITA

**Keywords:** anti-seizure medications, magnetic resonance imaging (mri), intravenous thrombolysis, seizure, stroke mimic

## Abstract

A wide range of acute neurological disorders may present with symptoms similar to a stroke, so-called 'stroke mimics'. Migraine aura and seizures account for the most extensive stroke mimics population. A large number of patients with a definite stroke mimics diagnosis (most commonly those with psychiatric disorders or seizures) had been treated with IV alteplase without adverse related events. We report a case of a man aged 70 years admitted to the emergency room because of acute onset of delirium and a loss of strength in the left arm (National Institutes of Health Stroke Scale {NIHSS}: 10), severe hyponatremia (127 mEq/L), and no evidence of intracranial arterial occlusion at CT scan. He was eligible for intravenous thrombolysis and, after treatment, neurological symptoms improved (NIHSS: 2). The subsequent appearance of “clonus” in the left lower limb, the persistence of hyponatremia, and the presence of electroencephalogram (EEG) abnormalities led to the clinical suspicion of focal motor-onset seizure with impaired awareness. The patient was treated successfully with anti-seizure medications (ASMs): lacosamide 200 mg IV during the acute setting care, followed by oral lacosamide 200 mg bis in die (BID). Since two other focal seizures occurred, brivaracetam 25 mg BID has been added in therapy with subsequent clinical discontinuance and EEG normalization. Two consecutive magnetic resonance imaging (MRI) examinations showed several cortical lesions restricted in high signal in diffusion‐weighted imaging (DWI) which corresponding to T2‐weighted and fluid‐attenuated inversion recovery (FLAIR) hyperintensities, but without lesions evidence in apparent diffusion coefficient (ADC) map. These radiological changes disappeared at a follow-up MRI performed 20 days after the symptoms’ onset. The patient fully recovered was discharged home without developing pharmacological adverse events. In this case, MRI provided an opportunity for early identification of seizure-related alterations. Hence, we discuss how prospective MRI studies during seizures and interictal period would contribute to defining the relationship between the electroclinical characteristics and MRI alteration patterns, and therefore, the potential role of MRI in the differential diagnosis between seizures and stroke mimic.

## Introduction

Twenty-five percent of direct admission to the hyperacute stroke unit (HSU) is represented by stroke mimics. The symptoms may underlie other medical or non-organic disorders (so-called 'medical’ or 'functional’ mimics, respectively). Several neurological or psychiatric disorders such as seizures, headache and migraine, decompensation, etc., in their early phases, can mimic ischemic stroke [1]. In the present study, we discuss a case of focal epilepsy with impaired awareness that mimicked ischemic stroke presenting with multiple acute MRI lesions, successfully treated with lacosamide and brivaracetam as the first add-on therapy. We believe this case report is both didactic and speculative since stroke is a time-related disorder and the differential diagnosis between stroke and stroke mimics is often hard in the emergency context, related to the lack of MRI images specificity and the several types of clinical presentation.

## Case presentation

**Figure 1:**
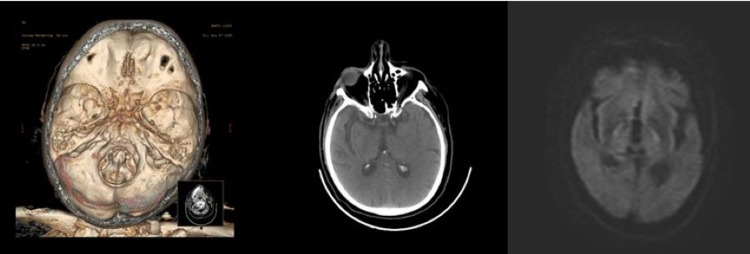
Volume-rendered image does not demonstrate any vessel occlusion. Focal abnormalities are not observed in the brain parenchyma at CT scan and DW images. DW: diffusion-weighted

We report a clinical case of a Caucasian man aged 70 years affected by major depressive disorder with psychotic symptoms home treated with mixed antipsychotic and antidepressant drugs, several different cerebrovascular risk factors (arterial hypertension, chronic obstructive pulmonary disease, obesity, and smoke), admitted to the emergency room because of acute onset of delirium, and a loss of strength in the left arm. Due to the pandemic, the clinical history was collected by telephone with relatives. The National Institutes of Health Stroke Scale (NIHSS) on ER admission was 10. Laboratory analysis revealed severe hyponatremia (127 mEq/L). CT angiogram of head and neck was obtained showing no arterial occlusion (Figure 1).

**Figure 2:**
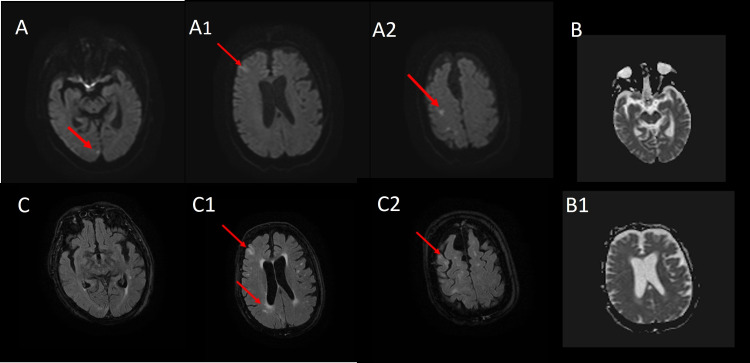
MRI acquired subsequently shows multiple right occipital, frontal, and parietal cortical lesions characterized by the high signal in DW images (A, A1, A2) and FLAIR (C, C1, C2), but without corresponding lesions on the ADC map (B, B1). FLAIR: fluid‐attenuated inversion recovery; DW: diffusion-weighted; ADC: apparent diffusion coefficient

The patient was eligible for intravenous thrombolysis with recombinant tissue plasminogen activator (r-tPA) and after treatment neurological symptoms improved (NIHSS: 2). A brain MRI was performed but did not highlight any acute ischemic lesions in diffusion-weighted imaging (DWI) (Figure 1). Diagnostic suspicion of focal motor-onset seizure with impaired awareness, according to the last International League Against Epilepsy (ILAE) classification, has been raised based on the late onset of clonus localized in the left lower limb combined with psychomotor agitation worsening, followed by Todd’s left paralysis [2]. Anti-seizure management with an IV bolus of lacosamide (200 mg infused in one minute) was administered with full recovery of neurological symptoms. The patient was admitted to the stroke unit for further investigations. Since extensive laboratory, cardiological, systemic vasculitis, and cancer screening were negative (except for hyponatremia), and the standard electroencephalogram (EEG) showed paroxysmal discharges in the right frontal and parietal hemisphere cortical regions, oral anti-epileptic therapy was started (lacosamide: 400 mg/day). During the 24 hours following hospitalization, two other focal seizures occurred. Hence, brivaracetam 25 mg BID has been added in therapy with subsequent clinical seizures discontinuance and EEG normalization. Two consecutive MRI examinations showed several cortical lesions restricted in high signal in diffusion‐weighted imaging (DWI) which corresponding to T2‐weighted and fluid‐attenuated inversion recovery (FLAIR) hyperintensities, but without lesions evidence in apparent diffusion coefficient (ADC) map (Figure 2).

**Figure 3:**
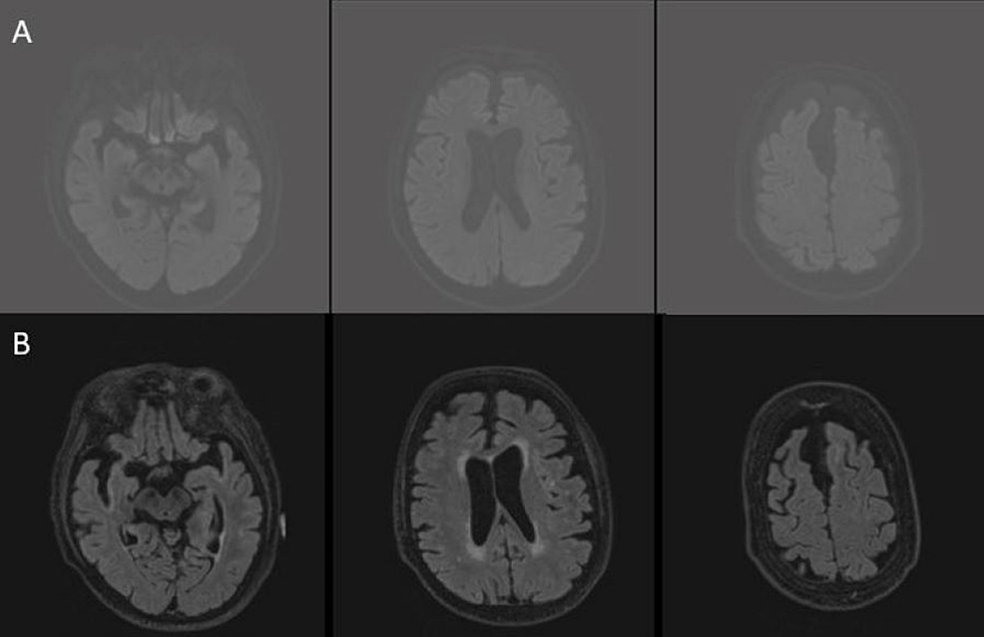
Follow-up MRI shows regression of the cortical lesions previously demonstrated both on DW (A) and FLAIR images (B). FLAIR: fluid‐attenuated inversion recovery; DW: diffusion-weighted

Transthoracic-transesophageal echocardiogram and 24-hour ECG monitoring were performed to rule out the presence of possible unknown embolic sources. The neuroimaging changes disappear at a follow-up MRI performed 20 days after the symptoms’ onset (Figure 3).

The persistent severe hyponatremia and a good therapeutic response to AEDs led to the definitive diagnosis of stroke mimic in acute onset of focal epilepsy with impaired awareness probably triggered by lasting electrolyte disturbance. However, the stroke mimics diagnosis and therapy management was complex, we left open further clinical hypotheses that have been averted through clinical and instrumental investigations (e.g., cerebrospinal fluid biomarkers, total body CT scan, blood cells autoimmunity panel, etc.). The patient fully recovered and was discharged home. At five weeks follow-up visit, the patient was seizure-free with behavioral fluctuation still persisting. Pharmacological adverse events were not detected.

## Discussion

Stroke mimic accounts for 25% of clinical conditions suspected of acute ischemic stroke and, among these, 72% present at least with one vascular risk factor and 15% suffer from functional disorder. Migraine aura and seizures include the most common neurological conditions that mimic stroke in the emergency setting. Since there are no specific guidelines for both functional or non-functional stroke mimics approach, identifying its presentation is the first step in improving its treatment and care. A significant number of stroke mimics are treated with alteplase in both retrospective studies and randomized controlled trials [3]. Thrombolysis can be administered even when a patient’s diagnosis is uncertain as it is relatively safe, especially compared with the risk of not treating a real stroke [4]. It has been observed that up to one-third of patients with minor stroke may show no evidence of DWI images restriction; indeed a negative MRI does not exclude the diagnosis of stroke [5]. MRI also provides an opportunity for early identification of seizures-activity related alterations. Variable peri-ictal MRI alterations (PMAs) have been reported in patients with status epilepticus (SE), both during the ictal and/or the postictal period. Restricted DWI sequences with corresponding low ADC and hyper‐intensities in T2‐weighted and FLAIR sequences that could even appear simultaneously are the most frequently encountered alterations. These changes represent a continuum of cytotoxic (increased DWI and decreased ADC signal) and vasogenic edema (increased DWI and increased T2 without decreased ADC signal) mostly depending on the timing of MRI execution [6,7]. These MRI seizure-related alterations are generally reversible; nevertheless, it is not clear how quickly they can normalize. Furthermore, in this reported case, the differential diagnosis among possible stroke mimics causes was more complex due to the simultaneous hyponatremia, probably drug-induced, which is the most common electrolyte disorder associated with stroke [8]. We considered appropriate the pharmacological association of the two latest-generation AEMs (lacosamide and brivaracetam) to obtain the best seizures control and also to avoid the negative interaction with the other drugs taken by the patient [9]. This patient suffered from the major depressive disorder with psychotic symptoms and among AEMs, brivaracetam seems to induce less impact on the psychotic sphere.

## Conclusions

This clinical case appears to be evocative, as it represents a typical neurological scenario in the emergency room. Acute ischemic stroke diagnosis is not obvious despite the possibility to access advanced neuroimaging technologies. Indeed, the differential diagnosis between stroke and stroke mimics is not always possible, especially since stroke is a time-related disease and treatment must be started as soon as possible. Especially, this clinical case was initially misleading since epilepsy occurred atypically. Probably, the possibility to perform an EEG in the emergency room could have guided the neurologist towards the correct diagnosis. Prospective MRI studies in patients with acute-onset epilepsy and positive neuroimaging would improve the diagnostic-therapeutic algorithm in doubtful cases between epilepsy and stroke.

## References

[1] Jones AT, O'Connell NK, David AS (2020). Epidemiology of functional stroke mimic patients: a systematic review and meta-analysis. Eur J Neurol.

[2] Fisher RS, Cross JH, French JA (2017). Operational classification of seizure types by the International League Against Epilepsy: position paper of the ILAE Commission for Classification and Terminology. Epilepsia.

[3] Khatri P, Kleindorfer DO, Devlin T (2018). Effect of alteplase vs aspirin on functional outcome for patients with acute ischemic stroke and minor nondisabling neurologic deficits: the PRISMS randomized clinical trial. JAMA.

[4] Tsivgoulis G, Alexandrov AV, Chang J (2011). Safety and outcomes of intravenous thrombolysis in stroke mimics: a 6-year, single-care center study and a pooled analysis of reported series. Stroke.

[5] Dawson A, Cloud GC, Pereira AC, Moynihan BJ (2016). Stroke mimic diagnoses presenting to a hyperacute stroke unit. Clin Med (Lond).

[6] Giovannini G, Kuchukhidze G, McCoy MR, Meletti S, Trinka E (2018). Neuroimaging alterations related to status epilepticus in an adult population: definition of MRI findings and clinical-EEG correlation. Epilepsia.

[7] Mendes A, Sampaio L (2016). Brain magnetic resonance in status epilepticus: a focused review. Seizure.

[8] Liamis G, Barkas F, Megapanou E (2019). Hyponatremia in acute stroke patients: pathophysiology, clinical significance, and management options. Eur Neurol.

[9] Steinig I, von Podewils F, Möddel G (2017). Postmarketing experience with brivaracetam in the treatment of epilepsies: a multicenter cohort study from Germany. Epilepsia.

